# Safety and tolerability of lenalidomide maintenance in post-transplant acute myeloid leukemia and high-risk myelodysplastic syndrome

**DOI:** 10.1038/s41409-021-01444-1

**Published:** 2021-09-01

**Authors:** Brian Pham, Rasmus Hoeg, Rajeev Krishnan, Carol Richman, Joseph Tuscano, Mehrdad Abedi

**Affiliations:** 1 Department of Hematology/Oncology, Davis Comprehensive Cancer Center, University of California, Sacramento, CA USA; 2 Department of Hematology/Oncology, Kaiser Northwest Permanente, Portland, OR USA

**Keywords:** Phase I trials, Acute myeloid leukaemia, Cancer therapeutic resistance

## Abstract

Relapse after allogeneic stem cell transplant in unfavorable-risk acute myeloid leukemia (AML) and high-risk myelodysplastic syndrome (MDS) portends a poor prognosis. We conducted a single-center phase I dose-escalation study with lenalidomide maintenance in high-risk MDS and AML patients after allogeneic transplantation. Sixteen patients enrolled in a “3 + 3” study design starting at lenalidomide 5 mg daily, increasing in increments of 5 mg up to 15 mg. Lenalidomide was given for 21 days of a 28-day cycle for a total of six cycles. Most common dose-limiting toxicities were lymphopenia, diarrhea, nausea, and neutropenia. Two patients had acute graft-versus-host disease (GVHD), and five patients developed chronic GVHD. The maximum tolerated dose was 10 mg, after dose-limiting toxicities were seen in the 15 mg group. Two dose-limiting toxicities were seen from development of acute GVHD and grade III diarrhea. Limitations of the study include time to initiation at 6 months post transplant, as many high-risk patients will have relapsed within this time frame before starting maintenance lenalidomide. Overall, lenalidomide was well tolerated with minimal GVHD and low rates of relapse rates, warranting further study.

## Introduction

Allogeneic stem cell transplantation (allo-SCT) remains the best option for cure in most patients with unfavorable-risk acute myeloid leukemia (AML) or high-risk myelodysplastic syndrome (MDS), but relapse and graft-versus-host disease (GVHD) after allo-SCT represent major challenges [1]. Approximately up to half of all patients with complex karyotype AML relapse after allo-SCT. In addition, secondary AML, age, active disease at time of transplant, and unfavorable cytogenetics are associated with increased risk of relapse [2]. GVHD represents the most common cause of mortality not related to disease relapse after transplant [3]. No standard treatment of post-transplant relapse exists. Options include salvage chemotherapy, donor lymphocyte infusion, and/or second transplant, but outcomes are very poor [4]. Strategies to decrease relapse after allo-SCT in this patient population of high-risk AML and MDS are needed. Maintenance therapy post transplant to enhance the graft-versus-leukemia (GVL) effect may be an effective strategy to prevent relapse [5]. As most relapses occur within 1 year of transplant, strategies to prevent early relapse could lead toward better survival outcomes [6].

Maintenance therapy after allogeneic transplant is a rapidly moving field, particularly in patients with targetable mutations. FLT3-positive patients clearly derive benefit from FLT3-directed maintenance, but the ideal drug for this is still being examined [7, 8]. Similarly, maintenance therapy for patients with IDH1 and IDH2 mutations is the topic of an ongoing clinical trial (NCT03564821). However, in patients without targetable mutations, the role for post-transplant maintenance is less clear. Hypomethylating agents have been studied for post allo-SCT maintenance therapy. Azacitidine and decitabine have been shown to increase GVL effect via upregulation and re-expression of epigenetically silenced genes. This leads to augmentation of CD8+ T cells, increasing cytotoxic effects upon tumor cells [9]. In addition, expansion of regulatory T cells modulates the GVHD effect. De Lima et al. demonstrated safety and tolerability of azacitidine after allo-SCT and found a 1-year overall survival of 86% [10]. Similarly, in a retrospective study, El Cheikh et al. found minimal toxicity and showed that 81% of patients remained in complete remission at ten months with post-transplant azacitidine maintenance [11]. Neither studies had graft rejection nor increased acute GVHD (aGVHD) incidence. Several phase I and retrospective studies have found similar outcomes; a phase III trial of azacitidine versus standard therapy was planned but ultimately closed due to slow accrual [12].

Lenalidomide, an immunomodulatory drug, is another candidate for potential maintenance therapy after allo-SCT [9, 10]. The mechanism of lenalidomide in AML and post-transplant maintenance is unknown. It has multiple effects on cytokines, immune cells, and angiogenesis. It has shown increased natural killer (NK) and cytotoxic T cell activity after transplant, as demonstrated in multiple studies [9, 13, 14]. Lenalidomide reduces TNF-alpha and T-regulatory cells that can lead to immune evasions by residual leukemic cells [15]. In addition, IL-12 production, cytotoxic T-cells, and NK cells are increased, each playing a specific role in the GVL effect [13, 16, 17]. All of this can be theorized to prevent relapse [13, 15] and in turn improve remission rates [10, 18].

However, Sockel et al. and Kneppers et al. reported higher incidence of aGVHD with lenalidomide as maintenance therapy after allo-SCT [19, 20]. Sockel et al. reported that 6/10 patients developed aGVHD after starting lenalidomide at a 10 mg daily dose, and four patients stopped therapy due to aGVHD. Kneppers et al. reported that 11/30 patients developed aGVHD and 5/30 developed extensive chronic GVHD (cGVHD) with lenalidomide as maintenance therapy after allo-SCT. The mechanism for this is not clear, given that lenalidomide is a derivative of thalidomide, which is used in cGVHD therapy after failed corticosteroid treatment [21]. The above studies started lenalidomide maintenance therapy early, within 6 months post allo-SCT. Early use of lenalidomide may enhance the GVHD more so than GVL. Therefore, during the design of our trial, the Federal Drug Administration advised starting lenalidomide no sooner than 6 months post allo-SCT to avoid potential aGVHD adverse events (AEs).

Our study was a prospective phase I safety and feasibility study of lenalidomide as maintenance therapy after allo-SCT for high-risk MDS and unfavorable AML patients. Secondary endpoints included overall survival, disease-free survival, and incidence of aGVHD/cGVHD.

## Methods

### Study design

This was a single-center, open-label phase I prospective study conducted in accordance with the Declaration of Helsinki. The institutional review board approved the study protocol. All study participants provided voluntary written informed consents. The study was registered on clinicaltrials.gov as NCT01433965.

### Patients

Patients aged 18–65 years with unfavorable-risk AML or high-risk MDS based on complex cytogenetics, refractory disease, secondary AML, residual disease at time of transplant, or International Prognostic Scoring System – Revised (IPPS-R) > 2 [18, 22, 23], who had undergone allo-SCT from mobilized peripheral bone marrow stem cells within 6–10 months prior to starting lenalidomide, were eligible. Patients needed to be in complete morphological remission and have Eastern Cooperative Oncology Group (ECOG) performance status score of 2 or lower. Exclusion criteria included use of azacitidine, decitabine, or other hypomethylating agents, lenalidomide, thalidomide, or pomalidomide after allo-SCT; active grade I or higher aGVHD during screening, concomitant malignancies, known hypersensitivity to thalidomide, donor chimerism less than 95%, pregnant or breast feeding females, or inability to sign informed consent.

### Endpoints

The primary endpoint was the tolerability and safety profile of lenalidomide in patients with unfavorable-risk AML or high-risk MDS post allo-SCT and to determine the maximum tolerated dose (MTD) of lenalidomide. Secondary endpoints included overall survival, relapse-free survival (RFS), and incidence of acute or cGVHD. Overall survival was defined as time from start of lenalidomide maintenance until death. RFS was defined as time from start of lenalidomide therapy until disease relapse, and aGVHD-free survival was defined as time from therapy until occurrence. cGVHD incidence was defined as worsening severity of pre-trial therapy and new onset cGVHD.

### Dose-escalation plan, safety, and tolerance

The study used a standard “3 + 3” dose-escalation design, starting at a dose of lenalidomide 5 mg daily and escalating to 10 and 15 mg daily. Lenalidomide was given daily for 21 days of a 28-day cycle for total for six cycles. Patients started treatment 6–10 months after transplantation [19, 20, 24]. MTD was defined as the dose at which 17% or less of (1 in 6) patients experienced dose-limiting toxicity (DLT). Toxicity grading was assessed via the National Cancer Institute Common Terminology Criteria for Adverse Effect Version 4.0 [25]. Severity of cGVHD is based on the National Institute of Health cGVHD consensus 2014 [26]. Definition of cGVHD and aGVHD was defined by the phenotypical presentation and biopsy as needed. Patients were monitored weekly in the first 4 weeks and every other week thereafter for AEs. DLTs were defined as non-hematological grade III–IV AEs and hematologic grade IV AEs not resolved after cessation of therapy within 7 days, aGVHD grade II–IV development while on therapy, or inability to continue therapy within the following 28-day cycle from toxicity related to treatment. If AEs occurred, lenalidomide was held until toxicity resolved and the dose was reduced by 5 mg. Therapy was discontinued in cases of disease progression, graft failure, grade II–IV aGVHD, no resolution of toxicity after 3 weeks of cessation for non-hematological grade III–IV toxicity or grade IV hematological toxicity, and inability to continue therapy within the following 28-day cycle due to an AE.

### Statistical analysis

Demographics, safety, and tolerability outcomes are reported in qualitative terminology. No direct comparisons are made among the dosing regimens. Overall survival was determined with the Kaplan–Meier method. Statistical analyses were performed with MedCalc version 18.10.2.

## Results

### Patient characteristics

Sixteen patients were enrolled into the study from February 2013 until May 2018; 13 had AML, 3 had MDS. Of the 13 AML patients, 7 had poor cytogenetics or FLT3 mutations, 7 had relapsed/refractory AML, and 5 had secondary AML.

Baseline characteristics are noted in Table 1 with associated transplant regimen. Three of the patients underwent salvage chemotherapy with fludarabine, high-dose cytarabine, and granulocyte colony stimulating factor (FLAG) followed by donor lymphocyte infusion after the preparative regimen. For the patients with MDS, all patients’ IPPS-R scores were greater than 4. In all, 25% (*n* = 4) of transplanted patients had matched sibling donors and the remaining 75% (*n* = 12) had matched unrelated donors. Median age was 54 (range, 34–64) with 32% (*n* = 5) over the age of 60 at time of study entry. Median time from allo-SCT until start of lenalidomide was 232 days (range; 180–315).

**Table 1:** Patient baseline characteristics.

Age	Gender	Disease type	Risk factors	BMT donor	Number of therapies before allo-SCT	Preparative regimen
63	F	AML	Complex cytogenetics	MUD	3	Bu4/Flu
62	M	AML	Secondary	MRD	1	Bu4/Flu
64	F	AML	Secondary	MUD	4	Bu2/Flu/ATG
36	M	AML	Relapsed/refractory	MRD	4	Bu4/Flu/FLAG+DLI
58	M	AML	Relapsed/refractory	MUD	4	Bu4/Flu/ATG
56	M	AML	Secondary, FLT3+	MUD	2	Bu4/Flu
34	M	AML	Secondary	MUD	1	Bu4/Flu/ATG
41	F	AML	Relapsed/refractory, complex cytogenetics	MUD	3	Bu4/Flu/FLAG+DLI
47	M	AML	Secondary, FLT3+, complex cytogenetics	MUD	2	Bu4/Flu/ ATG
44	M	AML	Relapsed/refractory, FLT3+	MUD	3	Bu4/Flu
54	M	MDS	IPSS-R score 5	MUD	1	Flu/Mel/ATG
63	F	MDS	IPSS-R score 5.5	MUD	1	Bu2/Flu
53	M	MDS	IPSS-R Score 6.5	MUD	1	Bu2/Flu
66	M	AML	Refractory/relapsed	MRD	4	Bu2/Flu/FLAG+DLI
54	F	AML	Relapsed/refractory, complex cytogenetics	MRD	4	Bu2Flu
29	F	AML	Relapsed/refractory, complex cytogenetics	MUD	5	Bu4/Flu

Bu2/Flu is a reduced intensive conditioning and Bu4/Flu is a myeloablative preparative regimen.

Bone marrow type donor: *MUD* matched unrelated donor, *MRD* matched related donor.

Conditioning regimens: *Flu* fludarabine, *Bu* busulfan, *Mel* melphalan, *ATG* antithymocyte globulin, *DLI* donor lymphocyte infusion, *FLAG* fludarabine, cytarabine, and granulocyte colony stimulating factor.

### Lenalidomide exposure

The median treatment duration for all patients was 131 days (range; 14–195) with 50% (*n* = 8) of patients completing the entire six cycles. Among the eight patients who completed the six cycles, four patients were exposed to 5 mg dosing, two patients exposed to 10 mg dosing, and two patients initially exposed to 15 mg but were subsequently dose reduced to 10 mg due to AEs. The dose reductions in the 15 mg group were due to fatigue and elevated LFTs, but both patients completed the six treatment cycles at 10 mg. Reasons for discontinuation were aGVHD (*n* = 2), patient withdrawal (*n* = 1), non-GVHD AEs (*n* = 4), and “other” (*n* = 1). The “other” was arthritis, which developed during therapy and decreased the patient’s quality of life; this resolved after cessation of therapy. The AEs leading to lenalidomide discontinuation were hospitalization for grade I diarrhea, nausea, and dehydration, causing a decrease in ECOG performance status, grade III diarrhea, grade IV neutropenia, and grade III thrombocytopenia. The grade IV neutropenia and grade III thrombocytopenia caused treatment delays of greater than 28 days. These toxicities all resolved after the cessation of lenalidomide.

### Safety and tolerability

The most common treatment-related adverse events (TRAEs) were related to gastrointestinal (GI) and hematological events. Table 2 reports the grade 1 or 2 TRAEs occurring in more than 10% of the patients and reports all grade 3 and 4 TRAEs. The most common grade 3 non-hematological TRAEs were diarrhea (*n* = 2, 12%) and nausea (*n* = 2, 12%). The most common hematologic grade 3 TRAEs were lymphopenia (*n* = 4, 25%) and neutropenia (*n* = 2, 12.5%). The only grade 4 TRAEs were neutropenia (*n* = 2, 12.5%) and leukopenia (*n* = 1, 6.25%). GI symptoms were the most common cause of discontinuation in this study; this included aGVHD and diarrhea. The most common hematological TRAE was lymphopenia. After dose reduction or cessation of the lenalidomide, the TRAEs resolved. There were no grade 5 TRAEs. In the 5 mg dosing group, one DLT occurred due to aGVHD; in the 10 mg dosing group, one DLT occurred due to grade 4 neutropenia causing treatment delay. In the 15 mg dosing group, there were two DLTs including one caused by aGVHD and another causing treatment delay due to grade III thrombocytopenia and diarrhea.

**Table 2:** Treatment-related adverse effects of lenalidomide maintenance therapy.

Adverse effects (total *n* = 16)	Grade 1–2	Grade 3	Grade 4
*Hematological effects*			
Lymphopenia	7	4	
Anemia	7	1	
Leukopenia	7		1
Neutropenia	4	2	2
Thrombocytopenia	4	1	
*Non-hematological effects*			
Fatigue	10		
Abdominal pain	7		
Alanine aminotransferase increased	7		
Diarrhea	6	2	
Alkaline phosphatase increased	5		
Rash, maculopapular	5		
Aspartate aminotransferase increased	4		
Constipation	4		
Pruritus	4		
Rash acneiform	4		
Anorexia	3		
Generalized muscle weakness	3		
Headache	3		
Nausea	2	2	
Vomiting	2	1	
INR increased		1	
Skin ulceration		1	

aGVHD was seen in two patients, presenting as GI aGVHD in both. Table 3 compares the development of GVHD in patients before and after treatment with the GVHD prophylaxis at time of transplant and enrollment of study. One patient’s acute GI GVHD was confounded by the cessation of GVHD prophylaxis 2 weeks prior to onset. The patient was placed back on GVHD prophylaxis and lenalidomide was stopped. While the aGVHD resolved, this patient later developed cGVHD involving the skin, liver, mouth, and eyes. The second case was grade 3 acute colonic GVHD and grade 2 acute esophageal GVHD. The onset of the second case was 2 weeks after starting lenalidomide 15 mg. The lenalidomide was stopped immediately at onset of symptoms. The patient was treated with prednisone and the GVHD resolved.

**Table 3:** GVHD prophylaxis during transplant and prior to initiation of lenalidomide with evaluation if patients were still on immunosuppression by end of the study.

GVHD prophyalxis during transplant	GVHD prophyalxis at start of maintenance	Off immunosuppression	GVHD prior to lenalidomide maintenance	GVHD after/during therapy
Cylosporine/mycophenolate/ATG	Cylcosporine	Yes	None	None
Tacrolimus/methotrexate	Tacrolimus	Yes	None	aGVHD grade IV gastrointestinal
Cyclosporine/mycophenolate	None	Yes	aGVHD grade I gastrointestinal	Mild cGVHD (liver)
Tacrolimus/methotrexate	None	Yes	None	Moderate cGVHD (skin, mouth)
Methotrexate/tacrolimus/ATG	Tacrolimus	Yes	Mild cGVHD (skin)	None
Tacrolimus/methotrexate	Tacrolimus	Yes	None	None
Tacrolimus/methotrexate	Tacrolimus	Yes	Moderate cGVHD (skin, oral, gastrointestinal)	None
Methotrexate/tacrolimus/ATG	Tacrolimus	Yes	Mild cGVHD (skin)	Mild cGVHD (mouth)
Tacrolimus/methotrexate	Tacrolimus	Yes	None	None
Tacrolimus/mycophenolate/ATG	Tacrolimus	Yes	Mild cGVHD (skin)	None
Cyclosporine/mycophenolate	Cyclosporine/prednisone	On sirolimus	Mild cGVHD (skin)	Mild cGVHD (skin)
Cyclosporine/mycophenolate/ATG	Cyclosporine	Yes	None	None
Cyclosporine /mycophenolate	Mycophenolate	Yes	None	None
Cyclosporine	Cyclosporine	Yes	Mild cGVHD (skin)	Mild cGVHD (eye)
Cyclosporine/mycophenolate	Cyclosporine/prednisone	Yes	None	None
Tacrolimus/mycophenolate	Tacrolimus/mycophenolate/prednisone	On tacrolimus	aGVHD grade I Liver	aGVHD grade III GI and grade II esophagus

GVHD before treatment and after lenalidomide maintenance therapy.

cGVHD developed in 31% (*n* = 5) of the patients but most resolved with immunosuppression. Most common cGVHD was skin (*n* = 2) and mouth (*n* = 2) with the highest severity as moderate. No patients discontinued the study after developing cGVHD. As seen in Table 3, four of five patients who developed cGVHD already had cGVHD prior to starting therapy. Only two patients still required immunosuppression at the end of follow-up. Of those two, one patient was still being treated with immunosuppression for aGVHD and the other for cGVHD.

### Disease relapse and overall survival

The median follow-up time of patients during the study was 1222 days (range; 375–1576). At the end of follow-up of the 16 patients, 2 had relapsed, while the 14 remained in remission as of October 2018. At 2 years, the estimated overall survival and the RFS is greater than 80%. One patient withdrew from the study. No secondary malignancy was observed after 1-year follow-up. One patient relapsed 340 days after completing the full six-cycle treatment with extramedullary disease (pelvic myeloid sarcoma) and subsequently passed away 768 days after relapse. The second patient relapsed 260 days after two cycles. This patient was unable to complete the full six cycles due to grade IV neutropenia. The patient subsequently passed away 563 days after relapse.

## Discussion

As relapse after allogeneic transplantation for AML and MDS is a major challenge, maintenance therapy should be explored in this population [27, 28]. There is currently no established role for maintenance after transplant in wild-type FLT3 AML. Hypomethylating agents and lenalidomide have been tested in this setting with mixed results. Particularly, the increased incidence of GVHD with the use of maintenance lenalidomide remains a concern [19, 20, 24].

Our study prospectively examined feasibility of post-transplant maintenance with lenalidomide in unfavorable-risk AML and high-risk MDS and found no increased incidence of GVHD. One explanation for this finding is the later (>6 months after transplant) start of lenalidomide, whereas previous studies have started earlier. Lenalidomide increases CD4+ and CD8+ T cells and IFN-y activity within weeks of starting therapy [9, 29]. This early induction of T-cell proliferation from the graft early post transplant may enhance aGVHD, whereas a later introduction of lenalidomide could avoid increased GVHD risk [20].

Only two occurrences of aGVHD occurred in our study; one was confounded by the withdrawal of GVHD prophylaxis 2 weeks previously. The second case of acute (grade 3 colonic) aGVHD resolved after cessation of lenalidomide and use of prednisone. No severe cGVHD was observed in the toxicity profile. None of the patients who developed cGVHD discontinued maintenance therapy. The survival outcomes and progression-free survival were generally favorable in this study, with only two patients experiencing relapse.

Lenalidomide maintenance therapy was generally well tolerated, and maximal dose tolerated reached in this study was 10 mg. At the 5 and 10 mg dosing regimen, there was one DLT. Most common TRAEs were GI and hematological. The 15 mg regimen had two DLTs, including aGVHD and grade III diarrhea. No patient in the 15 mg dosing regimen completed the full six-cycle maintenance therapy. Therefore, 10 mg dosing is the maximal tolerated dose to allow for completion of six cycles with the least amount of TRAE. As maintenance therapy post allo-SCT continues to be explored, there are no standards for timing of initiation or length of therapy. This study suggests that lenalidomide started 6 months at 10 mg after allo-SCT is well tolerated without increasing aGVHD. However, the length of six cycles requires further exploration.

The main limitations of this study and data were the small sample size and starting therapy in patients in complete remission 6 months post transplant. Choosing 6 months post transplant as the initiation time of therapy might make the use of lenalidomide safer and reduce incidence of aGVHD, but it invariably selects for a lower risk population, as many patients have relapsed at this point [8]. This would limit the applicability of maintenance to this specific subset population but this group of patients could still benefit as relapse between 6 months and 2 years portend to poor survival with estimated survival of 12% at 3 years. In addition, 39% of patients relapse between 6 months and 2 years [30]. Therefore, further investigations into phase II/III studies for lenalidomide would prove useful for this population subset.

In summary, this study demonstrates the feasibility and tolerance of lenalidomide after allo-SCT without apparent increase in acute or cGVHD. Ten milligram daily, given 21 of 28 days may be the regimen most likely to allow for completion of six cycles. Furthermore, the overall and progression-free survival were more than 80% at 2 years in this small group. These results warrant further larger prospective randomized clinical trials to explore the benefit of lenalidomide maintenance after allo-SCT for those with high-risk MDS and unfavorable AML.
